# Unraveling abundance from occurrence: Modeling an endangered rodent population with low capture probability

**DOI:** 10.1002/eap.70179

**Published:** 2026-02-11

**Authors:** Abby E. Bratt, Cheryl S. Brehme, Robert N. Fisher, Aaron J. Bertoia, Darryl I. MacKenzie

**Affiliations:** ^1^ Proteus Research and Consulting Ltd Outram New Zealand; ^2^ U.S. Geological Survey, Western Ecological Research Center San Diego California USA; ^3^ Present address: Nature Positive Dunedin New Zealand

**Keywords:** abundance prediction, Bayesian model, California, density estimation, endangered species, Heteromyidae, integrated model, occupancy modeling, Pacific pocket mouse, *Perognathus longimembris pacificus*

## Abstract

Predicting population abundance while accounting for uncertainty is an essential task for managers of endangered species but is often hindered by the challenge and expense of comprehensive data collection. Many traditional methods for estimating abundance of rare or elusive species are costly and logistically difficult, with occupancy‐based methods being a popular alternative. While the theoretical relationship between occupancy and abundance is well studied, there are few examples of methodological approaches for predicting abundance from occupancy. This study presents a novel approach to bridge the gap between abundance and occurrence for species with low capture probability, using the Pacific pocket mouse (*Perognathus longimembris pacificus*; PPM) in Southern California, USA, as a model system. PPM have been monitored across three subpopulations in this region using track tubes to inform occupancy over space and time and live captures to inform PPM demography and phenology. Paired capture–recapture data and presence–absence data collected between 2012 and 2022 were used to estimate density, occupancy, and detection, respectively. Density was modeled as a function of both occupancy and detection, and abundance at monthly and annual scales was predicted from estimates of density for all subpopulations. Our methodology leverages all available data in an integrated Bayesian analysis where uncertainty in site‐level abundance is naturally accounted for when scaling abundance estimates to the population level. While occupancy and detection were both predictive of and positively correlated with density, a meaningful amount of variation in density was not explained by our model, revealing avenues for future study as well as providing a realistic assessment of uncertainty in population‐level abundance predictions. In addition to advancing the current understanding of Pacific pocket mouse population dynamics, this approach is applicable to a wide array of species and ecosystems where population management is necessary, but individuals have low capture probability and available resources may preclude direct estimation of density at relevant spatial scales. From a design perspective, our results demonstrate the utility of strategically deploying density‐based monitoring methods within long‐term occupancy monitoring programs. More generally, our findings underscore the potential of this approach to inform methods to include abundance estimation in spatial occupancy monitoring programs for endangered species.

## INTRODUCTION

For managers tasked with the conservation of endangered species, accurately predicting population abundance and effectively characterizing the uncertainties associated with these estimates can be crucial for decision‐making. However, these tasks are frequently complicated by the significant difficulties and expenses associated with monitoring species that are rare or elusive, have large seasonal or annual variations in abundance, or occur over a large spatial extent (Emmet et al., 2021; Jones, 2011; MacKenzie et al., 2005; Thompson, 2004). The use of traditional sampling methodologies aimed at estimating the abundance of such species may be prohibited by low individual capture probabilities, high impacts to habitat and species, and high costs or logistical challenges (MacKenzie & Nichols, 2004). Instead, occupancy‐based sampling methods have emerged as a popular alternative. These methods, which estimate site occupancy from the presence or absence of species in a set of locations (MacKenzie et al., 2002, 2018), can offer a more feasible approach for continuous monitoring and assessment (MacKenzie & Nichols, 2004; Peralta et al., 2023; Stauffer et al., 2021). Despite the frequent use of occupancy estimates as indices of population abundance, the predictive relationship between occupancy and true abundance remains underexplored.

In the field of macroecology, abundance–occupancy relationships hypothesize that a more locally abundant species will occupy more sites than a less locally abundant species, and thus, the two quantities are positively correlated (Gaston et al., 2000; He et al., 2002; Ten Caten et al., 2022). While this relationship is often used to improve estimates of species distribution dynamics (e.g., Freckleton et al., 2006; He et al., 2002; Maclean et al., 2011), few examples use presence–absence data to predict true abundance (though see He & Gaston, 2000; Rossman et al., 2016; Royle & Nichols, 2003; Solow & Smith, 2010). Where many studies focus on the relationship between abundance and occupancy probability, Royle and Nichols (2003) posited that variation in abundance induces variation in occupancy detection probability, and produced a model that leverages the relationship between the two to estimate abundance. However, a consistent theme across studies is that the strength and shape of the relationships between occupancy probability, detection probability, and abundance are highly sensitive to species‐specific ecology (e.g., Buckley & Freckleton, 2010; Emmet et al., 2021; Linden et al., 2017), as well as the spatial and temporal sampling scales (Fuller et al., 2022; Hui & McGeoch, 2007; Steenweg et al., 2018; Wilson & Schmidt, 2015). Thus, we modeled the relationship between abundance and occupancy with a more flexible functional form than that of Royle and Nichols (2003). This is particularly important for species with low capture probability and for which conservative estimates of abundance are desirable.

Here, we present a unique case study of Pacific pocket mouse (*Perognathus longimembris pacificus*; PPM), a rare and elusive heteromyid in Southern California, USA (Brehme et al., 2023; Patton & Fisher, 2023). In this case study, annual widespread occupancy data were collected across several subpopulations and density data were collected at a subset of sites within each subpopulation at approximately the same spatial and temporal scales (Brehme et al., 2023). We introduce a novel method that leverages the relationship between occupancy, detection, density, and abundance to predict the latter from more easily acquired occupancy data. To our knowledge, our study is one of the first to explore the relationship between density and the combination of occupancy and occupancy detection (hereafter, detection) probabilities from long‐term field data.

We estimated two fitted relationships representing two temporal scales to capture different, but related, aspects of PPM ecology. At the monthly scale, density estimates represented a “snapshot” of PPM activity, operating under the assumption of demographic and geographic closure. In contrast, at the 4‐month scale (April–July), density estimates indexed cumulative PPM activity across their active season during which individuals may disperse, reproduce, and die. This dual approach allowed us to distinguish between immediate population status (monthly) and seasonal population dynamics (4‐month). We used both fitted relationships to predict abundance across multiple subpopulations to advance current knowledge of PPM population trends, reveal existing knowledge gaps in PPM ecology, and inform decision‐making surrounding future PPM monitoring and management.

The implications of this research extend beyond the immediate focus on PPM. By demonstrating the effectiveness of our method in a sparse data landscape typical of many endangered species, we highlight its potential applicability across a range of taxa and ecosystems. Moreover, our findings demonstrate that strategic use of traditional abundance monitoring techniques can complement long‐term occupancy monitoring programs when the goal is to better understand population dynamics as well as predict abundance over a larger landscape.

## METHODS

### Study system and species

**FIGURE 1:**
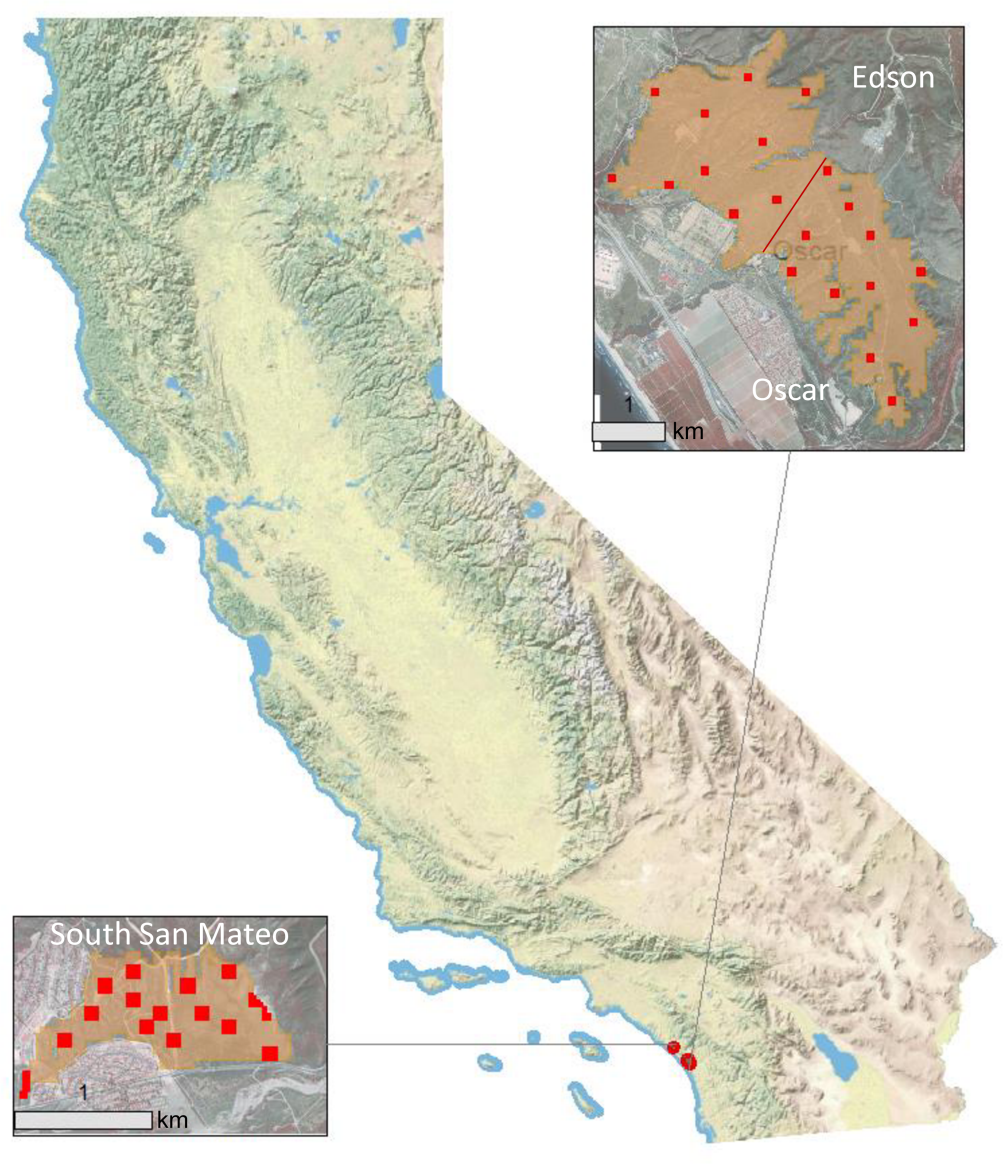
Pacific pocket mouse (PPM) mouse populations and monitoring areas on Marine Corps Base, Camp Pendleton (MCBCP). All squares represent permanent occupancy sampling plots with those annually live trapped shown in red. Yearly random plots are not shown. Base map from ESRI (2023).

The Pacific pocket mouse (*P. l. pacificus*; PPM) is one of 16 recognized subspecies of little pocket mouse (*Perognathus longimembris*; Patton & Fisher, 2023). Recently, it was shown that the PPM consists of two different morphologically divergent subspecies, *P. l. pacificus* and *Perognathus longimembris cantwelli*, with *P. l. pacificus* being restricted to the Tijuana Estuary Region and considered extinct, and *P. l. cantwelli* occurring across the remainder of the range including our populations studied here (Patton & Fisher, 2023). For consistency with most recent literature and pending genomic confirmation, we will continue to use *P. l. pacificus* sensu lato here. PPM were historically rare and patchily distributed along southern California marine terraces and alluvial plains within 4 km of the coast (Figure 1; Patton & Fisher, 2023). PPM are associated with sandy soils with low clay and silt content as well as with coastal sage scrub vegetation communities (Brehme et al., 2023). These habitats in southern California have been significantly reduced, leading to the subspecies being thought extinct in the 1970s, only to be rediscovered in 1993 and subsequently listed as endangered (Meserve, 1976; USFWS, 1994). PPM now face numerous threats and stressors including habitat loss and fragmentation, predation, competition for seed resources, and the impacts of human activity (USFWS, 1998).

PPM are nonsocial, nocturnal, and physiologically adapted to warm and dry climates. PPM live in burrows beneath the soil surface, where they go into variable amounts of facultative torpor during the late summer to winter and in response to low resource conditions. The onset of torpor is marked by a large drop off in activity that can occur from as early as June through November and is highly spatially variable within and among years (Brehme, Adsit‐Morris, et al., 2019; Meserve, 1976; Shier, 2009) with emergence typically occurring with increased resource availability in late winter to early spring (February–March; Vandergast et al., 2023). Average PPM home range size can vary spatiotemporally, with diameters between 13 and 24 m (Miller & Pavelka, 2008; Shier, 2009).

PPM demography has been shown to change within the active season, with the dominant demographic group transitioning from adult males to adult females to young of the year in line with reproductive activity (Brehme, Adsit‐Morris, et al., 2019; Miller & Pavelka, 2008; Shier, 2009). Breeding onset typically occurs in early spring following emergence from torpor. Females gestate young for approximately 3 weeks and wean young after approximately 30 days. In low‐resource years, reproduction may be skipped, while in high‐resource years, adult females may have up to three litters with their female offspring subsequently mating and reproducing within a single season (Miller & Pavelka, 2008). Shorter above‐ground activity has been documented to coincide with no or low reproduction (Brehme, Adsit‐Morris, et al., 2019). Because of these factors, PPM abundance can be highly variable both within and among years. PPM have a lifespan of up to 3–4 years in the wild (Bailey, 1939; French et al., 1967; Hayden & Lindberg, 1976).

There are currently three confirmed extant populations of PPM, two of which are on Marine Corps Base, Camp Pendleton, USA (MCBCP; USFWS, 1998, 2020; Figure 1). Within MCBCP, the two populations are split into three subpopulations based on large differences in habitat and management: (1) Santa Margarita: Oscar (411 ha); (2) Santa Margarita: Edson (474 ha); and (3) South San Mateo (105 ha; SSM) (Brehme, Adsit‐Morris, et al., 2019).

### Population monitoring data

Annual PPM monitoring has been conducted on MCBCP since 2012 for the primary purposes of (1) monitoring annual population status and trends in area occupied; and (2) understanding PPM occupancy, colonization, and extinction dynamics in relation to variables associated with landscape, soil, vegetation, climate, disturbance, and habitat management. For the monitoring design, sample plots were chosen probabilistically with respect to space and multiscaled with 64 0.0156‐ha subplots per 1‐ha plot. Subplots were monitored using track tubes (Brehme, Matsuda, et al., 2019), with each subplot the approximate size of a PPM core home range (Miller & Pavelka, 2008; Shier, 2009). Thirty‐three permanent plots were monitored from 2012 to 2017 and increased to 43 from 2018 to 2022 (Figure 1). An additional six randomly selected plots were sampled each year to increase spatial coverage over time. A subset of known‐occupied 0.25‐ha core plots within seven permanent grids were live‐trapped for five 3‐night sessions each year to collect temporal phenology data (reproduction and recruitment; Figure 1). Here, we leveraged the PPM capture data to inform estimates of PPM density at core plots.

#### Live‐trapping data

Live‐trapping data were collected at seven 0.25‐ha core plots annually from 2012 to 2022. The live‐trapping program was originally designed for the study of PPM reproductive phenology, and thus core plots were selected nonrandomly on the basis of known PPM occupancy. However, due to local extirpations, these included plots with no recorded PPM activity in some years. After 1–2 years of no PPM detections, locations for three plots were changed. In most years, five live‐trapping sessions were conducted at each of seven core plots, with most sessions occurring over the 4 months between April and July, when PPM are most likely to be active. Within each 0.25 ha core plot, live trapping was conducted on 16 0.0156 ha subplots (12.5 m × 12.5 m), using four traps per subplot. Although the total number of monitoring nights is substantially less than track‐tube monitoring, effort required was substantially greater for these data. Each trap‐night, three visits were required by a permitted biologist to each trap, resulting in nine visits per session and thus 45 visits annually (vs. 6–8 total track‐tube visits).

Live‐trapping sessions took place over 3 days, with traps being checked up to twice per day in the morning and night. Prior to each night of trapping, traps were baited and opened in the late afternoon, first checked in the late evening and again in the morning after which bait was removed and traps were closed. Captured PPM were assessed for age, sex, and reproductive condition. Individuals were marked with visible implant elastomer (VIE) tags in the base of the tail, such that they were individually identifiable upon recapture (Shier, 2009). For each 3‐night session, capture–recapture data were collated into individual encounter histories (i.e., sequences of 1's and 0's indicating whether an individual was captured in the respective occasion) at both the monthly and 4‐month temporal scales.

#### Track‐tube data

PPM tracks are easily distinguished from other sympatric rodents by size and shape, and thus track tubes were used to monitor PPM occupancy (Brehme, Matsuda, et al., 2019). The track tubes, made with 15″ polyvinyl chloride pipes, have ink pads at each end with track paper in between, baited with millet seed, and positioned on the ground where rodents can freely access and exit. When an animal enters the tube, the feet are inked, leaving tracks behind if the individual approaches the bait. The PPM track tubes and protocol are described in detail by Brehme, Adsit‐Morris, et al. (2019). Between 35 and 57 (mean: 46) 1‐ha plots were monitored with track tubes each year across the three subpopulations, with most (approx. 94%) plots being randomly selected. Six percent of plots throughout years were non‐randomly selected for the purpose of monitoring PPM responses to impacts, management, etc.

One track tube was placed in each 0.0156‐ha (12.5 m × 12.5 m) sampled subplot within a 1‐ha plot. This tube spacing approximates the PPM core home range, based on telemetry data (Shier, 2009). The proportion of the 1‐ha plot sampled using track tubes varied but was generally either 100% (64 tubes) or 50% (32 tubes) spaced evenly across the grid. Track tubes were deployed on all permanent and random plots continuously for approximately 4 months each year (e.g., April–July). Tubes were generally checked bi‐weekly, though the time between consecutive trap checks ranged from 7 to 21 days with 6–8 trap tube checks per 4‐month period. These detection–non detection data for PPM at each tube were collated into detection histories (i.e., sequences of 1's and 0's indicating whether PPM were detected in the respective occasion), at both the monthly and 4‐month temporal scales.

### Statistical modeling

By pairing live‐trapping sessions with track‐tube checks at core plots, there were sufficient data with which to estimate occupancy, detection probability, and density at these core plots. These paired estimates were then used to fit a relationship between occupancy, detection, and density (hereafter, hyper model) in an integrated analysis. This integrated analysis captured three data scenarios for a given 1‐ha plot across the population sites: (1) plot had both live‐trapping and track‐tube data; (2) plot had track‐tube data only; and (3) plot not sampled. In case (1), occupancy, detection, and density were estimated directly from data, and these estimates were jointly used to calibrate the hyper model in an integrated analysis. In case (2), occupancy and detection were estimated from track‐tube data, and the integrating hyper model was used to predict density from these estimates. In case (3), density was predicted based on estimates of density of representative plots (i.e., plots with similar features). In all cases, plot‐level abundances were predicted from expected densities and aggregated to the population level.

This process was repeated for each temporal scale, where the short scale was based on each of the calendar months of April–July and the longer 4‐month scale was based on aggregated data across April–July in each year from 2012 to 2022, creating two different time series of predicted population dynamics across the landscape where the monthly time series represents a series of “snapshots” of PPM activity and the 4‐monthly time series indexes cumulative PPM activity across the active season. The longer timescale, during which the population is not demographically or geographically closed, is included to account for (1) adults who may have been present, but not active above ground and thus not available for trapping at any given session due to facultative torpor; and (2) variable breeding phenology, with juveniles appearing above ground at different times across years.

There was not perfect spatial and temporal overlap of live‐trapping and track‐tube data at core plots, requiring some assumptions to be made to account for this mismatch. With respect to time, the track‐tube data used for the occupancy analysis encompassed a longer time period between checks than the 3‐day trapping session. We resolved this by defining both demographic parameters at the temporal scale of approximate calendar month, where live‐trapping sessions were associated with the calendar month in which the session occurred, and track‐tube data were associated with the calendar month in which the midpoint between trap checks occurred. With respect to space, both density and occupancy were estimated at the 1‐ha scale, though live traps sampled a smaller percentage of each 1‐ha core plot than the track tubes.

The following sections describe the model used for the short timescale. The general form of the model at the long timescale is the same, where aggregated data are used and the month effects are excluded. A detailed description of the 4‐month timescale model can be found in Appendix S1.

#### Density model

The live‐trapping data for PPM captured at least once were analyzed using the Huggins capture–recapture model (Huggins, 1991) to estimate capture probabilities and PPM abundance on the trapping grid.

For each core plot trapping month i, individual‐level capture histories were summarized to the number of unique individuals captured ($M_{i}$), total number of captures ($n_{i}$) and number of capture occasions ($t_{i}$). Capture probability ($p_{i}$) was assumed to vary according to the model

(1) $$\text{logit}\left(p_{i}\right)=\mu^{p}+\eta_{g_{i}}^{p}+\eta_{y_{i}}^{p}+\eta_{m_{i}}^{p}+\epsilon_{i}^{p},$$

where $\mu^{p}$ is the mean logit‐capture probability, $\eta_{g_{i}}^{p}$, $\eta_{y_{i}}^{p}$ and $\eta_{m_{i}}^{p}$ are random effects for the associated plot, year, and month, respectively and $\epsilon_{i}^{p}$ is a normally distributed random error term with mean equal to 0 and SD equal to $\sigma^{p}$. Each of the random effects was also assumed to be normally distributed with means equal to 0 and SDs of $\sigma_{g}^{p}$, $\sigma_{y}^{p}$, and $\sigma_{m}^{p}$, respectively.

The probability of an individual being captured at least once in plot trapping month i is defined as

(2) $$\begin{array}{c} p_{i}^{*}=1-{\left(1-p_{i}\right)}^{t_{i}}. \end{array}$$

PPM on a trapping array during the trapping is partitioned into two groups: (1) the number of animals captured at least once, $M_{i}$; (2) the number of animals never captured, $f_{i}$.

Under the Huggins' closed population capture–recapture model, the observed data likelihood for captured individuals was

(3) $$\begin{array}{c} L_{i}^{\mathrm{Hug}}=\frac{p_{i}^{n_{i}}{\left(1-p_{i}\right)}^{M_{i}t_{i}-n_{i}}}{{p_{i}^{*}}^{M_{i}}}. \end{array}$$

Partitioning the live‐trapping data in this way (i.e., into $M_{i}$ and $f_{i}$) enabled the mark–recapture data collected on those animals captured at least once to be analyzed using the Huggins model, which provides the necessary information on $p_{i}^{*}$ that is used to estimate $f_{i}$ and therefore $N_{i}$. Assuming that PPM abundance at plot‐month i, $N_{i}$, was a random variable from a Poisson distribution with expected value $\lambda_{i}$, it follows that

(4) $$\begin{array}{c} M_{i}˜\text{Poisson}\left(\lambda_{i}p_{i}^{*}\right), \end{array}$$

(5) $$\begin{array}{c} f_{i}˜\text{Poisson}\left(\lambda_{i}\left(1-p_{i}^{*}\right)\right), \end{array}$$

and

(6) $$\begin{array}{c} N_{i}=M_{i}+f_{i}, \end{array}$$

where the expected abundance at plot‐month i, $\lambda_{i}$, is related to the expected density, ${\overset{–}{D}}_{i}$, as well as the effective area sampled by the trapping array (A) according to,

(7) $$\begin{array}{c} \lambda_{i}={\overset{–}{D}}_{i}A. \end{array}$$

The effective area sampled in hectare, A, accounts for PPM movement and per Williams et al. (2002) was calculated as

(8) $$\begin{array}{c} A=L^{2}+4L\times 0.5d+\pi {\left(0.5d\right)}^{2}\times 10^{-4}, \end{array}$$

where L is the length of the sides of the trapping array (max. 37.5 m between traps on a 50 m side with 4 evenly spaced traps), and d is the diameter of a PPM home range, assuming circular home ranges for convenience. Values for d were drawn from a normal distribution to account for uncertainty in d, with mean (11.52 and 20.19 m at the short‐ and long timescales, respectively) and SD values (1.04 and 1.42 m, respectively) calculated from the maximum distance between trapping locations of individuals captured at least twice.

### Occupancy model

The proportion of sampled subplots occupied (PSO) by PPM, while accounting for imperfect detection, was estimated using the single‐season occupancy model of MacKenzie et al. (2002). As there were a limited number of sampled 0.0156‐ha subplots within a 1‐ha plot (i.e., 32 or 64 subplots), PSO was estimated as opposed to occupancy probability to account for the finite nature of the population of interest, where a substantial proportion of discrete spatial units were intensively surveyed. This approach, following MacKenzie et al. (2018), appropriately addresses the finite population correction needed when sampling from a known number of potential locations within defined plot boundaries.

The occupancy model was fit at the same temporal scale as the Huggins model where track‐tube checks roughly correspond to live‐trapping sessions, according to calendar month. For each plot‐month, i, subplot‐level track‐tube detection histories were summarized to the number of subplots surveyed ($s_{i}$), the number of subplots with at least 1 PPM detection ($s_{i}^{*}$), the number of PPM detections ($d_{i}$), and the number of trap checks ($k_{i}$). PPM detection probability ($\rho_{i}$) was assumed to vary according to the model

(9) $$\begin{array}{c} \text{logit}\left(\rho_{i}\right)=\mu^{\rho }+\beta^{\rho }X_{i}+\eta_{g_{i}}^{\rho }+\eta_{y_{i}}^{\rho }+\eta_{m_{i}}^{\rho }+\epsilon_{i}^{\rho }, \end{array}$$

where terms were defined and distributed analogously to the Huggins model, with the addition of $\beta^{\rho }$ which represents the effect of plot selection method X. Plot selection method X was a binary variable representing either randomly selected (X=0) plot or non‐randomly selected (X=1) plots (i.e., core plots, management grids, etc.). Note that $\rho_{i}$ is a species‐level detection probability, representing the probability that at least one individual is detected by the track tubes in plot‐month i, whereas $p_{i}^{*}$ defined above represents the probability that an individual is captured at least once by the live traps in plot‐month i. The probability of PPM occupancy ($\psi_{i}$) was assumed to vary according to the model

(10) $$\begin{array}{c} \text{logit}\left(\psi_{i}\right)=\mu^{\psi }+\beta^{\psi }X_{i}+\eta_{g_{i}}^{\psi }+\eta_{y_{i}}^{\psi }+\eta_{m_{i}}^{\psi }+\epsilon_{i}^{\psi }, \end{array}$$

where terms are defined analogously. The plot‐type coefficients were included to account for the hypothesis that occupancy and detection may be higher at nonrandomly selected plots.

PSO in plot‐month i, $\Psi_{i}$, was estimated as

(11) $$\begin{array}{c} \Psi_{i}=\frac{s_{i}^{*}+u_{i}}{s}, \end{array}$$

where $u_{i}$ is the number of subplots within the plot where PPM were present but undetected by tracking tubes, which was defined as

(12) $$\begin{array}{c} u_{i}˜\text{Binomial}\left(s-s_{i}^{*},\psi_{i}^{C}\right). \end{array}$$

$\psi_{i}^{C}$ is the probability of a subplot being occupied conditional on PPM not being detected there:

(13) $$\begin{array}{c} \psi_{i}^{C}=\frac{\psi_{i}{\left(1-\rho_{i}\right)}^{k_{i}}}{1-\psi_{i}\left(1-{\left(1-\rho_{i}\right)}^{k_{i}}\right)}. \end{array}$$

Under the MacKenzie et al. (2002) occupancy model, the observed data likelihood for each plot‐month was

(14) $$\begin{array}{c} L_{i}^{\mathrm{Mac}}=\psi_{i}^{s_{i}^{*}}\rho_{i}^{d_{i}}{\left(1-\rho_{i}\right)}^{s_{i}^{*}k_{i}-d_{i}}{\left(1-\psi_{i}\left(1-{\left(1-\rho_{i}\right)}^{k_{i}}\right)\right)}^{s-s_{i}^{*}}. \end{array}$$

### Hyper model

**FIGURE 2:**
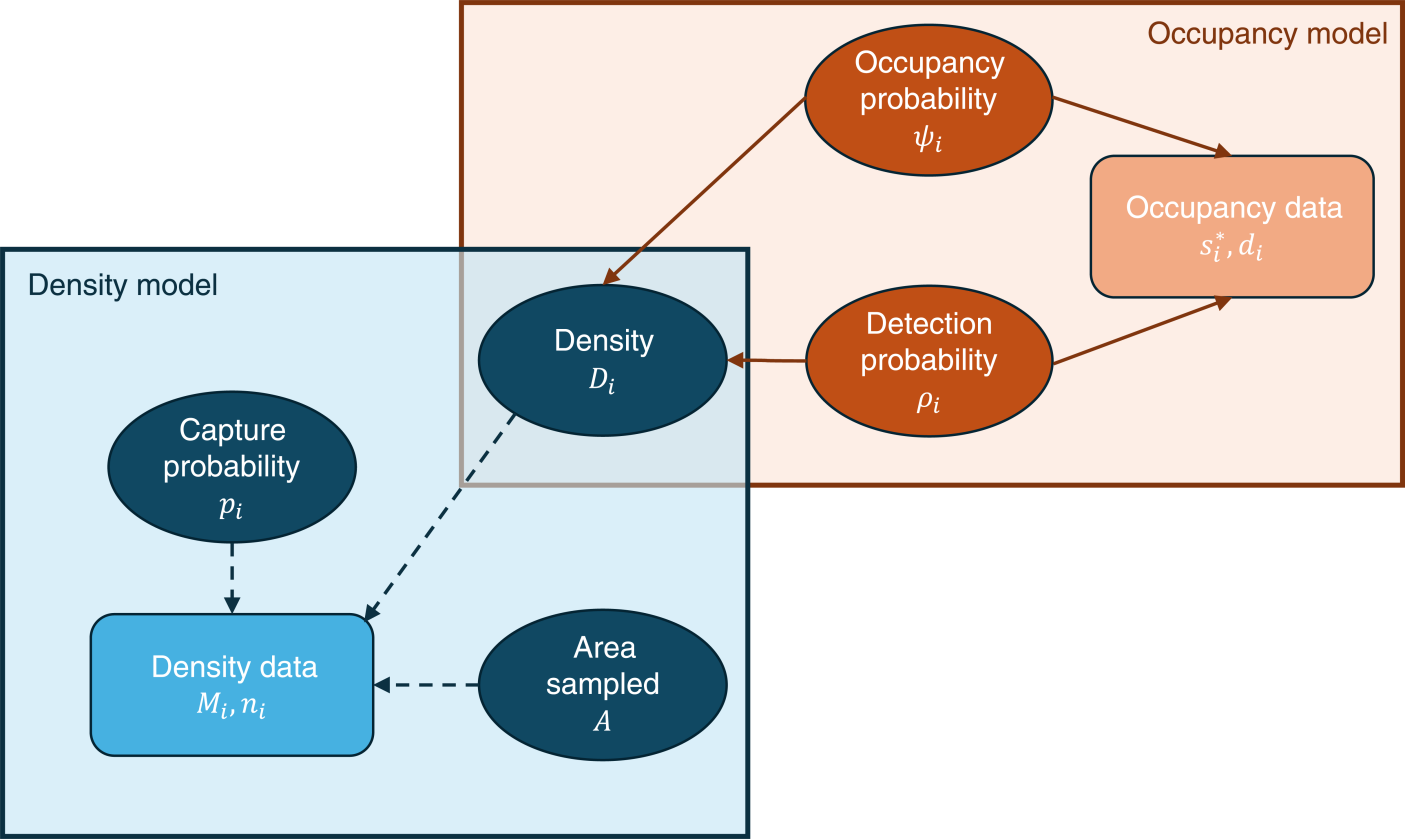
Simplified directed acyclic graph (DAG) of model, noting slight differences in approach by season length. Parameters are represented by ovals, with arrows pointing to boxes which represent the observed data arising from said parameters. The occupancy model is outlined in orange, while the density model is outlined in blue. As modeled, density is estimable from occupancy and detection and hence this parameter appears within both model outlines. An intermediate step in which Pacific pocket mouse (PPM) density and the area sampled yield on‐grid abundance (*N*) is omitted for simplicity. Also note that non‐random data values (e.g., number of sampling occasions) are omitted from this figure. Dashed lines indicate data case 2 (i.e., no capture data available); in case 1, all lines would be solid (i.e., contributing to joint estimation of density).

A “hyper model” was specified to model the relationship between expected PPM density, PSO, and track‐tube detection probabilities. The hyper model was essentially a regression equation where expected PPM density was defined as the response variable, and PSO and detection probability were predictor variables. Information about density, PSO, and detection probability was shared via the hyper model, which enabled the joint estimation of all parameters in a single integrated analysis (Figure 2).

The hyper model is defined as:

(15) $$\begin{array}{c} \log\left({\overset{–}{D}}_{i}\right)=\beta_{0}+\beta_{1}\sqrt{\Psi_{i}}+\beta_{2}\sqrt{\rho_{i}}+\epsilon_{i}, \end{array}$$

where ${\overset{–}{D}}_{i}$ is the expected PPM density at plot‐month i, $\Psi_{i}$ is the PSO at plot‐month i, $\rho_{i}$ is the probability of detecting PPM in a survey of an occupied subplot within a core plot during plot‐month i, and $\epsilon_{i}$ is a random error term distributed analogously to those above. The square‐root transformations of $\Psi_{i}$ and $\rho_{i}$ served dual purposes: (1) they created a model that was linear in terms of coefficients and transformed variables while ensuring a nonlinear relationship between $\log\left({\overset{⃐}{D}}_{i}\right)$ and the untransformed parameters (with the relative effect of $\Psi_{i}$ and $\rho_{i}$ on density decreasing as their values approach 1.0), and (2) it provided variance stabilization for these probability parameters. The log transformation of density was applied to normalize the assumed right‐skewed distribution of PPM density data that is common in other rodents (e.g., Singleton et al., 2005) and to stabilize variance.

### Model fitting

All analyses were conducted using Bayesian statistical methods, using the *JAGS* software, implemented from within *R* (R Core Team, 2023) using the *rjags* package (Plummer, 2023). Rather than defining the density and occupancy models in the *JAGS* code in terms of the latent and observed random variables, they were implemented using the “ones trick” (e.g., Kéry, 2010). That is, for a given data set, the observed data likelihood was calculated for the data obtained at trapping session i ($L_{i}$), and a vector of 1's was supplied as additional data ($x_{i}$). The $x_{i}$'s were mode modeled as Bernoulli random variables with success probability proportional to $L_{i}$. That is:

(16) $$\begin{array}{c} x_{i}˜\text{Bernoulli}\left(\gamma_{i}\right), \end{array}$$

where $\gamma_{i}=L_{i}/C$, and C is an arbitrarily large constant such that all $\gamma_{i}<1$. Implementing the models using this trick is more computationally efficient but mathematically equivalent to a state‐space formulation of the same model.

There were approximately 330 plot‐months (monthly scale) and 70 plot‐seasons (4‐month scale) with paired live‐trapping and occupancy observations to calibrate the hyper models. However, all occupancy data (approximately 2000 plot‐months, aggregated to approximately 500 plot‐seasons) were used to better inform estimates of occupancy and improve the predictive power of the calibrated model. Somewhat informative prior distributions were assumed for all parameters in the model based on expert opinion and as supported by preliminary analyses (Table 1). Truncated normal prior distributions were used for the σ parameters of the various random effect terms (ϵ and η) to represent that smaller values for σ were expected to be more likely a priori, and that while larger values were ecologically plausible, there was an upper bound on what values were considered plausible.

**TABLE 1:** Models for parameters within each sub‐model.

Model	Parameter	Prior	Timescale^a^
Hyper model	$\beta_{0}$	N(−3, 1)^b^	S, L
	$\beta_{1}$	N(0, 4)	S, L
	$\beta_{2}$	N(0, 4)	S, L
	σ	TN(0, 0.5625, 0, 1.5)^c^	S, L
Abundance	$\mu^{p}$	N(−1, 1)^b^	S, L
	$\sigma_{g}^{p}$	TN(0, 0.5625, 0, 1.5)^c^	S, L
	$\sigma_{y}^{p}$	TN(0, 0.5625, 0, 1.5)^c^	S, L
	$\sigma_{m}^{p}$	TN(0, 0.5625, 0, 1.5)^c^	S
	$\sigma^{p}$	TN(0, 0.5625, 0, 1.5)^c^	S, L
Occupancy	$\mu^{\rho }$	N(0, 1)	S, L
	$\beta^{\rho }$	N(0, 0.25)	S, L
	$\sigma_{g}^{\rho }$	TN(0, 0.5625, 0, 1.5)^c^	S, L
	$\sigma_{y}^{\rho }$	TN(0, 0.5625, 0, 1.5)^c^	S, L
	$\sigma_{m}^{\rho }$	TN(0, 0.5625, 0, 1.5)^c^	S
	$\sigma^{\rho }$	TN(0, 0.5625, 0, 1.5)^c^	S, L
	$\mu^{\psi }$	N(0, 1)	S, L
	$\beta^{\psi }$	N(0, 0.25)	S, L
	$\sigma_{g}^{\psi }$	TN(0, 0.5625, 0, 1.5)^c^	S, L
	$\sigma_{y}^{\psi }$	TN(0, 0.5625, 0, 1.5)^c^	S, L
	$\sigma_{m}^{\psi }$	TN(0, 0.5625, 0, 1.5)^c^	S
	$\sigma^{\psi }$	TN(0, 0.5625, 0, 1.5)^c^	S, L

*Note*: Where somewhat informative priors (starred) were used, support is given in the footnotes. Normal distributions are given with mean and variance and, for truncated normal distributions, the lower and upper bounds, respectively.

^a^ Timescales represented are the 1‐month/short (S) and 4‐month/long (L).

^b^ Weakly informative priors were used on intercept parameters for the hyper model and abundance components to improve sampling performance.

^c^ Truncated priors were used to constrain the variation between spatial and temporal units to ecologically plausible ranges based on preliminary analyses.

Posterior distributions for all parameters were approximated from three chains of 30,000 iterations with no thinning for the monthly scale and three chains of 300,000 iterations with every tenth iteration retained for the 4‐month scale. Thus, posteriors were approximated from 90,000 samples at both scales. Model convergence was evaluated with visual inspection of traceplots and the Gelman–Rubin statistic, $\hat{R}$, which was less than 1.1 for all parameters.

### Predicting population abundance

Predicting total PPM abundance at the three subpopulations was a primary goal of this analysis, with distinct approaches used for each of three data scenarios: (1) plot‐period i had paired live‐trapping and track‐tube data; (2) plot‐period i had only track‐tube data; and (3) no monitoring done in plot‐period i. In case (1), estimates of expected density from live‐trapping data were used directly. In case (2), track‐tube data informed estimates of occupancy which were then used in the integrated hyper model to generate density predictions. In case (3), predicted density for similar plot‐periods (i.e., same plot selection method, subpopulation, and year) were used; this case facilitates population‐wide abundance prediction. In all cases, abundance at the 1 ha plot scale was assumed to be a random variable from a Poisson distribution with expected value equal to the estimated or predicted density at a given plot‐period. A detailed explanation of how plot‐level abundance predictions were aggregated to the subpopulation‐level is given in Appendix S2.

## RESULTS

### Density model

The observed data were sparse, with the average number of unique individuals captured per plot‐month at the short timescale, $\bar{M},$ being 0.14 (SD = 1.1) and the average number of captures, $\bar{n}$, being 0.29 (SD = 2.3). At the long 4‐month scale, the average number of individuals captured per plot‐season was 1.19 (SD = 4.1) and the average number of total captures was 0.48 (SD = 2.0). The expected density, $\bar{D}$, across plots was 16.5 PPM/ha (90% credible interval: 0.6, 52.9) at the short timescale and 35.9 (1.8, 90.4) at the long timescale. The mean effective area sampled ($\bar{A}$) for each 0.25 ha core plot was 0.24 (0.22, 0.26) ha at the short timescale and 0.33 (0.30, 0.36) ha at the long timescale. At the short timescale, the mean capture probability, $\bar{p}$, for a single trap‐check was 0.05 (0.02, 0.21). Traps were checked 6 times per session, on average, which resulted in cumulative capture probability of 0.27 (0.1, 0.76). At the long timescale, the capture probability for a single trapping session was 0.08 (0.06, 0.11) and the cumulative capture probability was 0.29 (0.21, 0.39) over an average of 4 trapping sessions.

At the short timescale, the month and year random effects ($\sigma_{m}^{p}$ and $\sigma_{y}^{p}$, respectively; Appendix S3: Table S1) suggest substantial temporal variation in the capture probability. There was less spatial variation in capture probability, indicated by $\sigma_{g}^{p}$ (Appendix S3: Table S1). In contrast, at the long timescale, the spatial variation was greater than the temporal (i.e., between‐year) variation (Appendix S4: Table S1).

### Occupancy model

The proportion of surveyed subplots with at least one PPM detection ($s_{i}^{*}/s$) was approximately 0.14 (SD = 0.24) at the short (i.e., monthly) scale and 0.23 (0.33) at the long (i.e., 4‐month) scale. The expected occupancy probability, $\bar{\psi }$, across plots was 0.59 (90% credible interval: 0.08, 0.95) at the short timescale and 0.64 (0.08, 0.98) at the long timescale. There was no meaningful difference between occupancy by plot type at the short timescale (Appendix S3; Table S3). At the long timescale, there was evidence that occupancy was higher at nonrandomly selected plots (Appendix S4; Table S3), though the effect was small ($\beta^{\psi }=0.59;-0.1,1.29$).

At the short timescale, the mean detection probability, $\bar{\rho }$, for a single tube check was 0.47 (0.04, 0.87). Tubes were checked twice per month, on average, which resulted in a cumulative detection probability of 0.72 (0.08, 0.98). At the long timescale, the detection probability for a single tube check was 0.40 (0.06, 0.75) and the cumulative capture probability was 0.97 (0.32, 0.99) over an average of seven checks. As with occupancy probability, there was not a meaningful difference in detection by plot type at the short timescale (Appendix S3: Table S2), while there was some evidence at the long timescale that detection was higher at nonrandomly selected plots (Appendix S4: Table S2), although the effect was small ($\beta^{\rho }=0.28;-0.4,0.97$).

For both ψ and ρ, there was a considerable level of spatial, temporal, and remaining unexplained variation at both timescales (Appendix S3: Tables S2 and S3 and Appendix S4: Tables S2 and S3). For ρ, the spatial variation between plots was much greater than any temporal variation at both timescales.

### Hyper model

**FIGURE 3:**
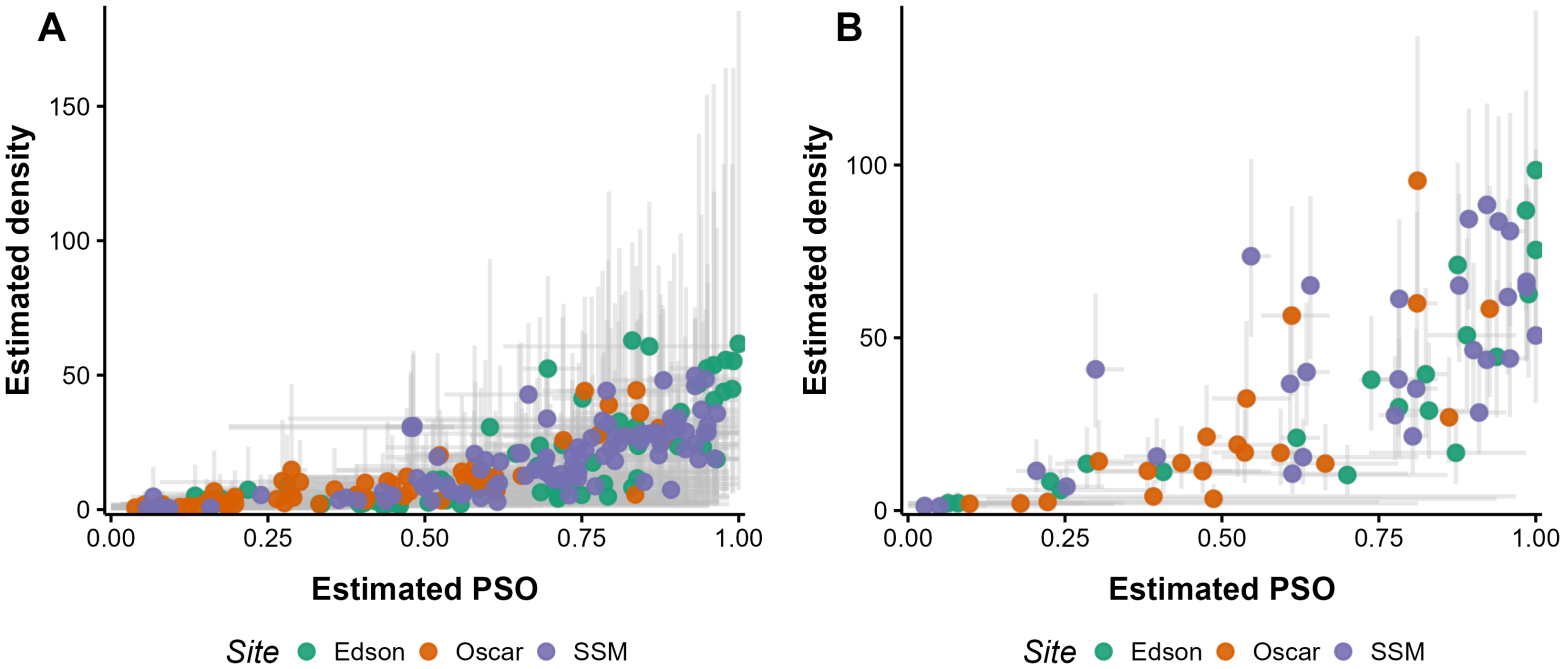
Estimated relationship between expected Pacific pocket mouse (PPM) density (per hectare) and proportion of subplots occupied (PSO) by PPM, according to the short timescale model (A) and long timescale model (B). Points indicate the posterior mean, and error bars indicate 90% credible intervals. Points are colored by subpopulation. SSM, South San Mateo.

**FIGURE 4:**
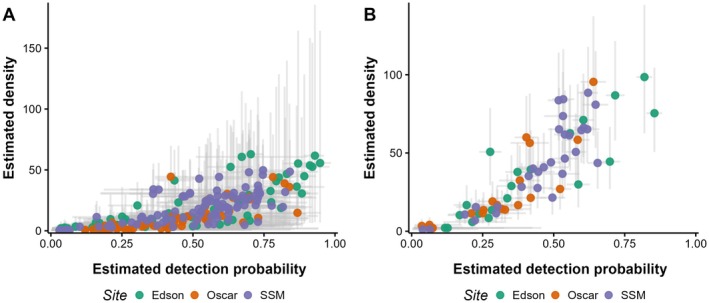
Estimated relationship between expected Pacific pocket mouse (PPM) density (per hectare) and PPM detection probability, according to the short timescale model (A) and long timescale model (B). Points indicate the posterior mean, and error bars indicate 90% credible intervals. Points are colored by subpopulation. SSM, South San Mateo.

Summaries of the posterior distributions for the hyper‐model parameters at both timescales are given in Table 2. The magnitude of the posterior distributions for $\beta_{0}$ indicated that expected density was small when the estimated PSO and probability of PPM detection were low. PSO and detection were strong positive predictors of expected density at both timescales (Figures 3 and 4). The magnitude of the SD of the random error term on the scale of log‐density for the hyper‐model ($\sigma_{\epsilon }$; Table 2) suggests that there was additional variation in expected density not explained by PSO and detection.

**TABLE 2:** Summary of posterior distributions for hyper‐model parameters at the short‐term scale.

Parameter	Short timescale			Long timescale		
	Mean	SD	90% CrI	Mean	SD	90% CrI
$\beta_{0}$	−2.47	0.64	(−3.54, −1.44)	−0.92	0.44	(−1.67, −0.2)
$\beta_{1}$	3.15	0.87	(1.75, 4.62)	2.15	0.68	(1.02, 3.27)
$\beta_{2}$	3.14	0.93	(1.6, 4.64)	3.88	0.79	(2.58, 5.17)
$\sigma_{\epsilon }$	0.94	0.16	(0.7, 1.23)	0.48	0.08	(0.35, 0.61)

*Note*: Given are the posterior mean, SD and limits of a 90% credible interval (CrI).

### Predicted population abundance

**FIGURE 5:**
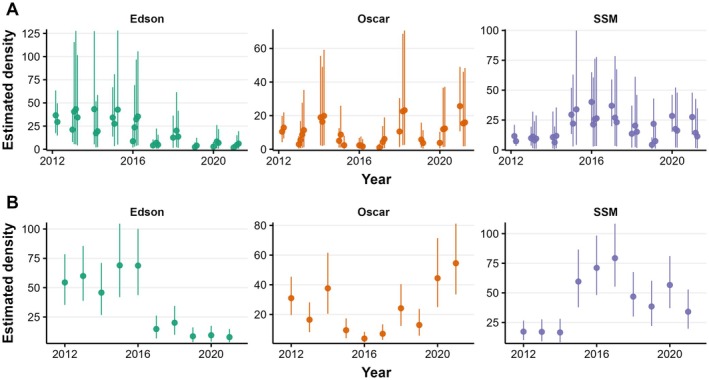
Estimated expected Pacific pocket mouse (PPM) density (per hectare) according to the short timescale model (A) and long timescale model (B). Points indicate the posterior mean, and error bars indicate 90% credible intervals. SSM, South San Mateo.

**FIGURE 6:**
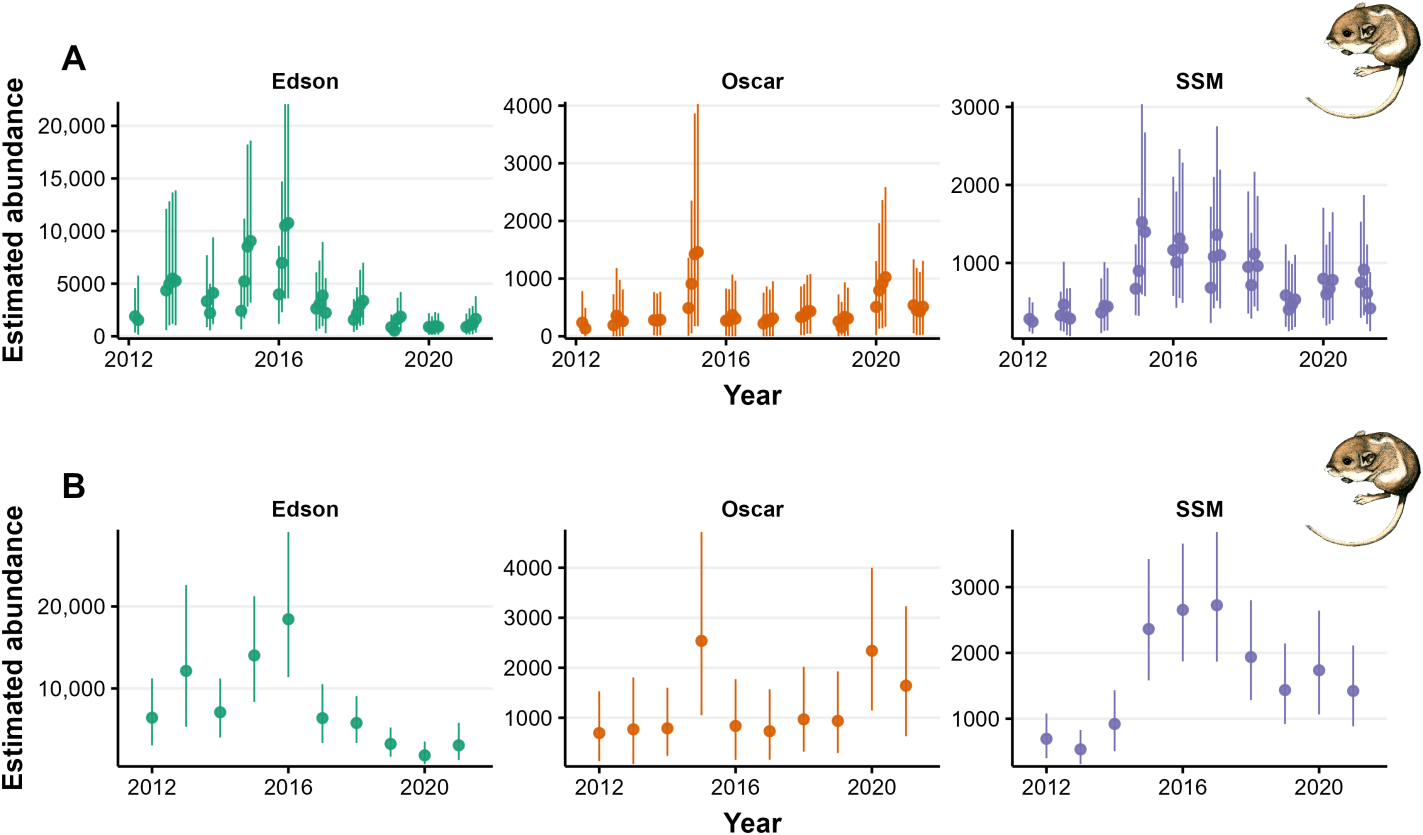
Estimates of abundance across the data period for each Pacific pocket mouse (PPM) subpopulation, according to the short timescale model (A) and long timescale model (B). Points indicate the posterior mean, and error bars indicate 90% credible intervals. PPM artwork by Tristan Edgarian. SSM, South San Mateo.

Over the study period, predicted abundance across all subpopulations at the long timescale ranged from a high of 21,931 in 2016 to a low of 5,612 in 2019. Predicted abundance in any given month, using the short timescale model, was smaller (Figures 5A and 6A). In general, predicted abundance increased over the season; at the short timescale, the average population growth rate between months was 1.16, underscoring that the population is not closed at the 4‐month scale and estimates should be interpreted as indexing cumulative activity. Similarly, monthly estimates may underrepresent PPM abundance due to periods of inactivity for a proportion of the population. Annual abundance predictions from the long timescale model are compared to the abundance predictions from the short timescale model for the end of each monitoring year (i.e., July) in Table 3.

**TABLE 3:** Summary of predicted abundance across Pacific pocket mouse subpopulations and years, presented for July only, using the short‐term model.

Site	Year	Short timescale			Long timescale		
		Mean	SD	90% CrI	Mean	SD	90% CrI
Edson	2012	1540	2546	(147, 5771)	6434	2488	(3059, 11,232)
	2013	5269	5353	(1034, 13,869)	12,142	6112	(5326, 22,621)
	2014	4114	3131	(1131, 9401)	7097	2424	(4017, 11,205)
	2015	9057	5598	(3200, 18,606)	14,026	4119	(8358, 21,250)
	2016	10,766	6795	(3604, 22,069)	18,441	5423	(11,385, 29,083)
	2017	2230	2373	(291, 5525)	6384	2178	(3355, 10,519)
	2018	3369	2256	(1098, 6983)	5790	1745	(3363, 9063)
	2019	1865	1585	(443, 4216)	3239	1147	(1676, 5236)
	2020	905	760	(148, 2180)	1840	892	(772, 3529)
	2021	1650	1301	(340, 3811)	3071	1493	(1272, 5808)
	2022	1791	1313	(433, 4053)	4189	1655	(2104, 7179)
Oscar	2012	133	184	(0, 492)	695	494	(132, 1530)
	2013	260	322	(8, 811)	769	548	(73, 1805)
	2014	285	285	(17, 771)	788	543	(239, 1600)
	2015	1459	1545	(173, 4026)	2537	1160	(1050, 4715)
	2016	305	355	(0, 965)	836	527	(158, 1772)
	2017	311	359	(0, 952)	732	471	(161, 1570)
	2018	434	342	(42, 1079)	969	593	(321, 2018)
	2019	308	317	(8, 843)	937	562	(293, 1927)
	2020	1024	1023	(165, 2587)	2340	985	(1145, 3998)
	2021	510	440	(25, 1308)	1642	829	(632, 3231)
	2022	861	720	(196, 1990)	1266	551	(549, 2384)
SSM	2012	246	151	(94, 494)	695	214	(400, 1080)
	2013	286	223	(60, 677)	535	166	(310, 831)
	2014	440	289	(135, 936)	921	299	(506, 1432)
	2015	1398	778	(574, 2672)	2363	589	(1582, 3426)
	2016	1191	647	(489, 2286)	2654	584	(1872, 3660)
	2017	1099	640	(417, 2194)	2724	638	(1868, 3837)
	2018	960	561	(385, 1857)	1937	474	(1283, 2801)
	2019	531	339	(178, 1104)	1436	395	(921, 2143)
	2020	781	534	(266, 1649)	1736	506	(1066, 2643)
	2021	418	274	(125, 883)	1422	385	(885, 2113)
	2022	1161	658	(453, 2309)	2188	567	(1415, 3206)

*Note*: Given are the mean, SD, and 90% credible interval (CrI) of the posterior distributions.

Abbreviation: SSM, South San Mateo.

On average, abundance was highest at Edson and lowest at Oscar, although density was highest at SSM and lowest at Oscar (Figures 5 and 6). Between‐year abundance trends varied between sites, with the Edson and SSM subpopulations peaking in 2016 followed by a population decline, while the Oscar population stayed relatively steady over the data period.

## DISCUSSION

This study introduces a novel methodological framework for fitting a relationship between species density and species occupancy and detection probability. Using a case study of PPM in southern California, USA, we demonstrate both the underlying methodology and use the calibrated relationship to predict abundance across multiple subpopulations, while accounting for uncertainty. Our findings underscore the potential of this general approach for broader application across various species and ecosystems, and particularly for species or environments in which capture probabilities are low. However, we emphasize that because occupancy–abundance relationships are sensitive to underlying species biology as well as the spatial and temporal scales of sampling, the general approach presented here should be adapted to suit different contexts on a case‐by‐case basis. In this case, PPM are largely solitary with very small home ranges and the spatial scale of sampling within the plots was consistent with their core home range size. The temporal scale of sampling was dictated by logistical constraints, and thus two timescales were considered to capture both short‐term and long‐term measures of abundance.

The difference in spatiotemporal sampling scales for the track‐tube and live‐trapping data in this study underscores the need for careful alignment of the scales of data collection in future applications if the goal is to estimate abundance accurately. Previous studies have identified several ways in which occupancy relationships are sensitive to temporal sampling scales. In particular, the nonlinearity of the abundance–occupancy relationship increases with sampling duration (Fuller et al., 2022; Steenweg et al., 2018) as well as sampled area (He & Gaston, 2008; Webb et al., 2019), both of which were observed in this study. Moreover, the relationship exhibits heteroskedasticity (i.e., variance in abundance not constant with respect to occupancy) that is dependent on both sampling duration (Steenweg et al., 2018) and sampling area (He & Gaston, 2008). Thus, differences between the scales of abundance data collection and occupancy data collection could impact the predictive ability of the relationship in unexpected ways and should be addressed on a case‐by‐case basis. Here, we managed differences in spatiotemporal scales by placing constraints on both the shape of the relationship and the coefficients. The model form, where density on the logarithmic scale was a linear function of root occupancy and root detection, was decided a priori. Given that this model was developed to inform the management of an endangered species, we made analysis decisions that erred towards conservative (i.e., lower) abundance estimates. Modeling occupancy and detection as functions of plot selection method, under the hypothesis that occupancy and detection probabilities were higher at non‐randomly selected plots, was one of these decisions; accounting for this effect avoids overestimating abundance. At the same time, the functional form of the model allows expected density to be nonzero even in the event of estimated PSO and detection probabilities of zero given that it has an intercept term. We also made several simplifying assumptions, including that PPM are homogeneously distributed over the landscape; these assumptions could be relaxed in future applications.

PPM have long been a challenging species to monitor due to low probabilities of capture in live‐traps, as shown in this study and Miller and Pavelka (2008), likely exacerbated by variable above‐ground activity of individuals (Miller & Pavelka, 2008). Live trapping over extended periods is very costly, results in high impacts to vulnerable habitats, and increases stresses to PPM from confinement and handling. In comparison, tracking tubes that continuously monitor PPM presence and temporal distribution require only a single visit every 2–3 weeks and thus large numbers of track tubes can be run for long periods at relatively low cost, including through unpredictable temporal periods where variable proportions of PPM are active. At the smallest scale of the subplot, which was designed to be the size of a PPM core home range, our estimates of abundance trend closely with area occupied, showing that occupancy (at the 0.0156 ha spatial scale) and abundance are closely correlated for this species (Brehme, Adsit‐Morris, et al., 2019). Abundance estimates for informing decisions on population translocations are desired by conservation agencies to increase the number of PPM populations. These analyses allowed us to estimate both monthly and annual abundances and can be used conservatively as an important tool for making translocation trapping decisions without altering the design of the current monitoring program.

The credible intervals around predictions of population‐wide abundances are notably wide. These wide intervals primarily reflect the sparse nature of the data with relatively few captures and detections per session, which may be expected consequences of PPM demography, behavior, and phenology. While the wide intervals serve as an indicator of where additional data collection could most improve precision in future research, they also underscore the benefit of an integrated approach. That is, abundance estimates based on the small number of plots with live‐trapping data alone would be associated with even greater uncertainty. This effect is most pronounced for species with low capture probabilities.

While occupancy and detection are both strong predictors of abundance, there was a large amount of unexplained variation in density. In addition to low capture probabilities, other knowledge gaps may be targeted by future monitoring and analysis. In the case of PPM, a 2‐stage design where all occupied plots were later trapped would be extremely costly and likely not possible to do within a reasonable time frame. However, with our model, focused trapping for an increased number of nights and plots in July, when density and detection probabilities are highest, may provide a more precise estimate if desired, but potentially at the cost of more specific data on annual reproductive phenology and recruitment. Therefore, any change in sampling will depend on whether the goals or scope of the monitoring program are changed. Without any change, our model provides a tool for continued prediction of abundance from occupancy data.

PPM habitat is highly dynamic, and the availability of suitable habitat at occupied sites is highly variable among years, depending on landscape and environmental conditions (e.g., vegetation, rainfall, and wildfire) and habitat management (e.g., prescribed burns; Brehme et al., 2023). Currently, some of the most predictive covariates from field surveys of sample plots include cover of non‐native grasses (negatively associated with PPM occupancy; Brehme et al., 2023) and forbs (positively associated with PPM occupancy), which are indistinguishable in most available remotely sensed data, and complete sampling of annual vegetation data across population areas is costly to collect on the ground. However, we expect that integrating additional spatial covariates or habitat suitability models that cover the entire areas of inference may better explain variation in both occupancy and density of unsampled plots for this model and help to produce more precise population‐level abundance estimates.

While our study represents a significant step forward in the application of occupancy data for population abundance prediction, particularly for species with low individual capture probabilities, it also highlights the complexities and nuances of ecological modeling. The effective implementation of this method will require ongoing refinement, rigorous validation, and a nuanced understanding of its limitations. This study paves the way for simulation and evaluation of the model's performance across different data scenarios and species. In particular, future work could test this method on a dataset for which density estimation was the primary goal and for which this is reflected in the sampling design. Such research is vital to understanding the limits and strengths of the proposed methodology and its application, to further validate its reliability and sensitivity in varied ecological contexts. For PPM, we were able to utilize occupancy data from the extant monitoring program and produce conservative abundance estimates to be utilized in the future management of this species. This is a cost‐effective and low‐impact alternative to conducting additional live trapping given the low probability of individual capture. More generally, by integrating density and occupancy estimates through a hyper model, we offer an approach to utilize presence–absence data to predict abundance across broad spatial and temporal scales in cases where traditional methods may be cost‐prohibitive, destructive, or otherwise infeasible.

## AUTHOR CONTRIBUTIONS

Cheryl S. Brehme and Robert N. Fisher contributed to the design and implementation of the research. Abby E. Bratt and Darryl I. MacKenzie contributed to the design and implementation of the analytical approach. Abby E. Bratt, Cheryl S. Brehme, Robert N. Fisher, Aaron J. Bertoia, and Darryl I. MacKenzie all contributed critically to the manuscript.

## CONFLICT OF INTEREST STATEMENT

The authors declare no conflicts of interest.

## Supporting information


Appendix S1.

Appendix S2.

Appendix S3.

Appendix S4.

## Data Availability

Data supporting this research are restricted and not available publicly. Capture‐ and detection‐histories are owned by Marine Corps Base, Camp Pendleton, USA, and available to qualified researchers by contacting James Asmus, Uplands Management Section Head, Environmental Security, at james.asmus@usmc.mil and requesting capture‐ and detection‐histories from Pacific pocket mouse monitoring on Marine Corps Base, Camp Pendleton between 2012 and 2022. Code (Bratt & MacKenzie, 2025) is available in Zenodo at https://doi.org/10.5281/zenodo.17567323.
