# Supplementary material for: Unraveling abundance from occurrence: Modeling an endangered rodent population with low capture probability
Source: Ecol Appl. 2026 Feb 11;36(1):e70179. doi: 10.1002/eap.70179 (PMC12892172; doi:10.1002/eap.70179)
Supplement: Supplementary file 1 — Appendix S1. [file EAP-36-e70179-s004.pdf]

Unraveling abundance from occurrence: Modeling an endangered rodent population with low capture probability

Ecological Applications

Abby E. Bratt, Cheryl S. Brehme, Robert N. Fisher, Aaron J. Bertoia, Darryl I. MacKenzie

Appendix S1: Statistical model at 4-month scale

The general forms of the density and occupancy models at the 4-month scale were similar to the 1-month scale. Small adjustments were made to account for the aggregated data and to exclude the monthly effects, with the exact details given below. The form of the hyper-model was identical across temporal scales; we refer readers to the main text for details.

Density model

As in the monthly model, the live-trapping data for PPM captured at least once were analyzed using the Huggins capture-recapture model Huggins (1991) to estimate capture probabilities and PPM abundance on the trapping grid.

For each core plot trapping season i, individual-level capture histories were summarized to the number of unique individuals captured (M_i), the total number of captures in trapping session j within the 4-month season (n_{ij}), the number of capture occasions within trapping session j (t_{ij}), and the number of capture sessions within the 4-month season (T_i). Capture probability (p_i) was assumed to vary according to the model

$$\text{logit}(p_i) = \mu^p + \eta^p_{g_i} + \eta^p_{y_i} + \epsilon^p_i,$$

where μ^p is the mean logit-capture probability, $\eta^p_{g_i}$, and $\eta^p_{y_i}$ are random effects for the associated grid and year, respectively, and ϵ^p_i is a normally distributed random error term with mean equal to 0 and standard deviation equal to σ^p. Each of the random effects were also assumed to be normally distributed with means equal to 0 and standard deviations of σ^p_g, and σ^p_g, respectively.

Variation in the number of capture occasions within trapping sessions was accounted for by defining the trapping session capture probability, P_{ij}, as

$$P_{ij} = 1 - (1 - p_i)^{t_{ij}},$$

where t_{ij} is the number of capture occasions within trapping session j at plot-season i. The probability of at least one capture in the entire 4-month trapping season, i, was

$$p^*_i = 1 - \prod_{j=1}^{T_i} \left(1 - P_{ij}\right).$$

where T_i is the number of trapping sessions within plot-season i.

As in the monthly model, PPM on a trapping array during the trapping are partitioned into two groups: (1) the number of animals captured at least once, M_i; (2) the number of animals never captured, f_i. Both quantities were assumed to follow Poisson distributions as in the monthly model, with total abundance (i.e., $M_i + f_i$)

$$N_i \sim \text{Poisson}(\lambda_i),$$

where λ_i is a function of the expected density, $\bar{D}_i$, as well as the effective area sampled by the trapping array (A). That is,

$$\lambda_i = \bar{D}_i A.$$

The effective area sampled in ha, A, accounted for PPM movement and per Williams et al. (2002) was calculated as

$$A = L^2 + 4Ld + \pi d^2 \cdot 10^{-4},$$

where L is the length of the sides of the trapping array (max. 37.5 m between traps on a 50 m side with 4 evenly spaced traps), and d is the radius of PPM homerange. Values for d were drawn from a normal distribution to account for uncertainty in d. The mean and standard deviation values (20.19 m and 1.42 m, respectively) were calculated from the maximum distance between trapping locations of individuals captured at least twice over the 4-month timescale.

Under the Huggins closed population capture-recapture model with constant capture probability, the observed data likelihood was

$$L_i^{Hug} = \frac{\prod_{j=1}^{T_i} P_{ij}^{n_{ij}} \left(1 - P_{ij}\right)^{M_i t_i - n_{ij}}}{p_i^{*M_i}}.$$

Partitioning the live-trapping data in this way enables the mark-recapture data collected on those animals captured at least once to be analyzed using the Huggins model, which provides the necessary information on p_i^* that is used in combination with the density hyper-model, to estimate f_i and therefore N_i.

Occupancy model

The proportion of sampled subplots occupied (PSO) by PPM, while accounting for imperfect detection of PPM, was estimated using the single-season occupancy model of MacKenzie et al. (2002). As there were a limited number of sampled 0.015625 ha subplots within a 1 ha plot (i.e.,

32 or 64 subplots), PSO was estimated to account for the finite nature of the population of interest (i.e., subplots within a plot; MacKenzie et al. 2018).

For each plot-season, i, subplot-level track tube detection histories were summarized to the number of subplots surveyed (s_i), the number of subplots with at least 1 PPM detection (s_i^*), the number of PPM detections (d_i), and the number of trap checks over the 4-month season (k_i). PPM detection probability (ρ_i) was assumed to vary according to the model

$$\text{logit}(\rho_i) = \mu^\rho + \beta_i^\rho X_i + \eta_{g_i}^\rho + \eta_{y_i}^\rho + \epsilon_i^\rho,$$

where terms are defined and distributed analogously to the Huggins model, with the addition of β^ρ which represents the effect of grid selection method X. Grid selection method X was a binary variable representing either randomly selected ($X = 0$) grids or non-randomly selected ($X = 1$) grids (i.e., core plots, management grids, etc.). The probability of PPM occupancy (ψ_i) was assumed to vary according to the model

$$\text{logit}(\psi_i) = \mu^\psi + \beta^\psi X_i + \eta_{g_i}^\psi + \eta_{y_i}^\psi + \epsilon_i^\psi,$$

where terms are defined analogously.

PSO in plot-season i, Ψ_i, was estimated as

$$\Psi_i = \frac{s_i^* + u_i}{s},$$

where u_i was the number of subplots within the plot where PPM were present but undetected by tracking tubes, which was defined as

$$u_i \sim \text{Binomial}(s - s_i^*, \psi_i^C).$$

ψ_i^C was the probability of a subplot being occupied conditional on PPM not being detected there

$$\psi_i^C = \frac{\psi_i(1-\rho_i)^{k_i}}{1-\psi_i(1-(1-\rho_i)^{k_i})}.$$

Under the MacKenzie et al. (2002) occupancy model, the observed data likelihood for each plot-season was

$$L_i^{Mac} = \psi_i^{s_i^*}\rho_i^{d_i}(1-\rho_i)^{s_i^* k_i - d_i}\left(1-\psi_i(1-(1-\rho_i)^{k_i})\right)^{s-s_i^*}.$$

References

Huggins, R.M. 1991. Some Practical Aspects of a Conditional Likelihood Approach to Capture Experiments. Biometrics 47 (2): 725–32.

MacKenzie, D.I., J.D. Nichols, G.B. Lachman, S. Droege, J.A. Royle, and C.A. Langtimm. 2002. Estimating site occupancy rates when detection probabilities are less than one. Ecology 83 (8): 2248–55.

MacKenzie, D.I., J.D. Nichols, J.A. Royle, K.H. Pollock, L.L. Bailey, and J.E. Hines. 2018. Occupancy estimation and modeling: Inferring patterns and dynamics of species occurrence. Second edition. Elsevier, Amsterdam; Boston.

Williams, B.K., J.D. Nichols, and M.J. Conroy. 2002. Analysis and management of animal populations. Academic Press, San Diego, CA.
