# Supplementary material for: Unraveling abundance from occurrence: Modeling an endangered rodent population with low capture probability
Source: Ecol Appl. 2026 Feb 11;36(1):e70179. doi: 10.1002/eap.70179 (PMC12892172; doi:10.1002/eap.70179)
Supplement: Supplementary file 2 — Appendix S2. [file EAP-36-e70179-s003.pdf]

Unraveling abundance from occurrence: Modeling an endangered rodent population with low capture probability

Ecological Applications

Abby E. Bratt, Cheryl S. Brehme, Robert N. Fisher, Aaron J. Bertoia, Darryl I. MacKenzie

Appendix S2: Predicting population abundance

The procedure used to predict population abundance at the subpopulation scale aggregated predictions made from each of three data scenarios: (1) plot-period i had paired live-trapping and track-tube data; (2) plot-period i had only track-tube data; and (3) no monitoring done in plot-period i. In case (1), estimates of expected density from live-trapping data were used directly. In case (2), track-tube data informed estimates of occupancy which were then used in the integrated hyper-model to generate density predictions. In case (3), predicted density for similar plot-periods (i.e., same grid selection method, subpopulation, and year) were used in a bootstrap-like approach; this case facilitates population-wide abundance prediction. In all cases, abundance at the 1 ha plot scale was assumed to be a random variable from a Poisson distribution with expected value equal to the estimated or predicted density at a given plot-period.

To adequately characterize uncertainty in predictions of abundance (N_i) in each plot-period with data (i.e., cases 1 and 2), we sampled 10,000 values of N_i from its posterior distribution. For plot-periods with live-trapping data, we also sampled 10,000 values of A from its posterior distribution to account for uncertainty in the effective area sampled by each trapping session. For the 1-month timescale and within each subpopulation (S), year (y), month (m), and grid selection method (X), posterior samples were summarized by taking

$$A^{*}_{S,y,m,X,a} = \sum_{g=1}^{G_{S,y,m,X}} A_{S,y,m,X,g,a},$$

where G represents the number of sampled grids within the relevant subpopulation, year, month, and type and a indexes each of the 10,000 posterior samples. The proportion of the total subpopulation area sampled, $A^{\phi}_{S,y,m,X,}$, was then taken by dividing $A^{*}_{S,y,m,X}$ by $A^{G}_{S,X}$, the total area of type X in subpopulation S. Similarly, the predicted abundance on sampled plots within each subpopulation, year, month, and grid selection method (X) was summarized by taking

$$N^{*}_{S,y,m,X,a} = \sum_{g=1}^{G_{S,y,m,X}} N_{S,y,m,X,g,a}.$$

Month-specific predictions are aggregated to the subpopulation-level according to

$$N^{total}_{S,y,m,a} = \sum_{X} \left(\frac{N^{*}_{S,y,m,X,a}}{A^{\Phi}_{S,y,m,X,a}} \right).$$

Scaling up abundance estimates in this way retains uncertainty captured in the posteriors, while accounting for variation in abundance between subpopulations and across years, months, and plot types, even for unsampled plots as in data case 3.

An alternative method for predicting expected density and expected abundance at unsampled plots was explored, in which independent predictions of $N_{S,y,m,X,g,a}$ were generated for all unsampled 1 ha plots directly from the posteriors for underlying model parameters. However, given the relative uncertainty in each posterior, this approach resulted in credible intervals that were impractical for species management. The approach described above results in tighter credible intervals, whilst still incorporating the observed variability in occupancy and abundance.

Predictions at the 4-month timescale were made analogously, excluding the month effects.
