# Supplementary material for: Unraveling abundance from occurrence: Modeling an endangered rodent population with low capture probability
Source: Ecol Appl. 2026 Feb 11;36(1):e70179. doi: 10.1002/eap.70179 (PMC12892172; doi:10.1002/eap.70179)
Supplement: Supplementary file 3 — Appendix S3. [file EAP-36-e70179-s001.pdf]

Unraveling abundance from occurrence: Modeling an endangered rodent population with low capture probability

Ecological Applications

Abby E. Bratt, Cheryl S. Brehme, Robert N. Fisher, Aaron J. Bertoia, Darryl I. MacKenzie

Appendix S3: Additional results, 1-month scale

Table S1: Summary of posterior distributions for capture probability (p) parameters at the short timescale. Given are the posterior mean, standard deviation (SD) and limits of a 90% credible interval (CrI).

Parameter	Mean	SD	90% CrI
μ^p	-2.91	0.77	(-3.9, -1.51)
σ^p	0.20	0.13	(0.02, 0.44)
σ_g^p	0.65	0.24	(0.32, 1.12)
σ_m^p	1.35	0.13	(1.1, 1.49)
σ_y^p	1.41	0.09	(1.25, 1.49)

Table S2: Summary of posterior distributions for PPM detection probability (ρ) parameters for the short timescale. Given are the posterior mean, standard deviation (SD) and limits of a 90% credible interval (CrI).

Parameter	Mean	SD	90% CrI
μ^ρ	-2.30	0.40	(-2.9, -1.63)
σ^ρ	1.46	0.04	(1.38, 1.5)
β^ρ	0.00	0.50	(-0.83, 0.82)
σ_g^ρ	1.48	0.02	(1.43, 1.5)
σ_m^ρ	0.41	0.25	(0.13, 0.93)
σ_y^ρ	0.64	0.21	(0.32, 1.01)

Table S3: Summary of posterior distributions for PPM occupancy probability (ψ) parameters, for the short timescale. Given are the posterior mean, standard deviation (SD) and limits of a 90% credible interval (CrI).

Parameter	Mean	SD	90% CrI
μ^ψ	-1.96	0.41	(-2.6, -1.27)
σ^ψ	1.49	0.01	(1.46, 1.5)
β^ψ	0.00	0.50	(-0.82, 0.82)
σ_g^ψ	1.48	0.02	(1.45, 1.5)

Parameter	Mean	SD	90% CrI
σ_m^{ψ}	0.27	0.21	(0.03, 0.7)
σ_y^{ψ}	0.93	0.23	(0.58, 1.33)

Table S4: Summary of predicted abundance at Edson site across months and years using the short-term model. Given are the mean, SD, and 90% credible interval (CrI) of the posterior distributions.

Site	Year	Month	Mean	SD	90% CrI
Edson	2012	Jun	1890	2365	(352, 4575)
Edson	2012	Jul	1540	2179	(147, 5772)
Edson	2013	Apr	4354	5156	(587, 12112)
Edson	2013	May	4989	6010	(1032, 12839)
Edson	2013	Jun	5479	6735	(1185, 13673)
Edson	2013	Jul	5269	6333	(1035, 13870)
Edson	2014	May	3324	4054	(861, 7706)
Edson	2014	Jun	2201	2775	(555, 4998)
Edson	2014	Jul	4114	5045	(1131, 9401)
Edson	2015	Apr	2417	3031	(661, 5378)
Edson	2015	May	5222	6396	(1682, 11183)
Edson	2015	Jun	8529	10344	(2805, 18229)
Edson	2015	Jul	9058	11034	(3201, 18606)
Edson	2016	Apr	3981	4949	(1178, 8601)
Edson	2016	May	6975	8546	(2281, 14717)
Edson	2016	Jun	10512	12901	(3566, 22066)
Edson	2016	Jul	10766	13292	(3604, 22070)
Edson	2017	Apr	2629	3322	(513, 6076)
Edson	2017	May	3043	3846	(721, 7200)
Edson	2017	Jun	3870	4870	(963, 8970)
Edson	2017	Jul	2231	2768	(292, 5525)
Edson	2018	Apr	1573	1964	(421, 3528)
Edson	2018	May	2138	2669	(604, 4655)
Edson	2018	Jun	2993	3694	(984, 6305)
Edson	2018	Jul	3370	4148	(1099, 6983)
Edson	2019	Apr	868	1127	(143, 2068)
Edson	2019	May	540	873	(0, 2026)
Edson	2019	Jun	1634	2067	(418, 3662)
Edson	2019	Jul	1865	2349	(443, 4217)
Edson	2020	Apr	892	1160	(140, 2198)
Edson	2020	May	751	991	(134, 1894)
Edson	2020	Jun	949	1219	(150, 2296)
Edson	2020	Jul	906	1175	(149, 2180)
Edson	2021	Apr	881	1148	(160, 2163)
Edson	2021	May	1064	1377	(164, 2660)
Edson	2021	Jun	1155	1481	(174, 2866)
Edson	2021	Jul	1650	2086	(341, 3812)

Site	Year	Month	Mean	SD	90% CrI
Edson	2022	Apr	1466	1859	(287, 3517)
Edson	2022	May	2010	2514	(543, 4502)
Edson	2022	Jun	2181	2724	(602, 4748)
Edson	2022	Jul	1792	2263	(433, 4054)

Table S5: Summary of predicted abundance at SSM site across months and years using the short-term model. Given are the mean, SD, and 90% credible interval (CrI) of the posterior distributions.

Site	Year	Month	Mean	SD	90% CrI
Oscar	2012	Jun	236	302	(40, 783)
Oscar	2012	Jul	133	119	(0, 492)
Oscar	2013	Apr	192	255	(0, 729)
Oscar	2013	May	356	506	(0, 1182)
Oscar	2013	Jun	293	472	(0, 978)
Oscar	2013	Jul	261	328	(8, 812)
Oscar	2014	May	281	389	(16, 766)
Oscar	2014	Jun	270	381	(9, 742)
Oscar	2014	Jul	285	397	(17, 771)
Oscar	2015	Apr	489	686	(9, 1363)
Oscar	2015	May	910	1196	(51, 2353)
Oscar	2015	Jun	1425	1768	(175, 3865)
Oscar	2015	Jul	1459	1809	(173, 4026)
Oscar	2016	Apr	272	394	(8, 827)
Oscar	2016	May	270	392	(0, 817)
Oscar	2016	Jun	370	557	(0, 1070)
Oscar	2016	Jul	306	402	(0, 965)
Oscar	2017	Apr	220	370	(0, 758)
Oscar	2017	May	281	398	(0, 863)
Oscar	2017	Jun	261	389	(0, 809)
Oscar	2017	Jul	312	417	(0, 953)
Oscar	2018	Apr	334	458	(16, 865)
Oscar	2018	May	352	477	(17, 904)
Oscar	2018	Jun	432	569	(45, 1060)
Oscar	2018	Jul	435	574	(42, 1080)
Oscar	2019	Apr	257	358	(0, 727)
Oscar	2019	May	155	240	(16, 596)
Oscar	2019	Jun	336	463	(0, 936)
Oscar	2019	Jul	308	407	(9, 843)
Oscar	2020	Apr	510	679	(16, 1304)
Oscar	2020	May	795	991	(132, 1962)
Oscar	2020	Jun	914	1136	(138, 2362)
Oscar	2020	Jul	1025	1278	(165, 2587)
Oscar	2021	Apr	537	701	(50, 1335)
Oscar	2021	May	453	606	(15, 1184)
Oscar	2021	Jun	430	571	(17, 1117)

Site	Year	Month	Mean	SD	90% CrI
Oscar	2021	Jul	511	684	(26, 1308)
Oscar	2022	Apr	574	746	(98, 1468)
Oscar	2022	May	650	832	(111, 1627)
Oscar	2022	Jun	581	756	(107, 1415)
Oscar	2022	Jul	862	1085	(197, 1991)

Table S6: Summary of predicted abundance at SSM site across months and years using the short-term model. Given are the mean, SD, and 90% credible interval (CrI) of the posterior distributions.

Site	Year	Month	Mean	SD	90% CrI
SSM	2012	Jun	282	341	(121, 560)
SSM	2012	Jul	247	259	(94, 495)
SSM	2013	Apr	327	395	(133, 633)
SSM	2013	May	465	583	(122, 1015)
SSM	2013	Jun	307	394	(75, 674)
SSM	2013	Jul	287	365	(61, 677)
SSM	2014	May	365	447	(102, 802)
SSM	2014	Jun	452	552	(130, 1011)
SSM	2014	Jul	441	550	(136, 936)
SSM	2015	Apr	669	790	(329, 1239)
SSM	2015	May	899	1090	(322, 1834)
SSM	2015	Jun	1522	1846	(595, 3036)
SSM	2015	Jul	1398	1689	(574, 2672)
SSM	2016	Apr	1165	1366	(578, 2104)
SSM	2016	May	1009	1212	(425, 1916)
SSM	2016	Jun	1313	1577	(550, 2459)
SSM	2016	Jul	1191	1440	(489, 2286)
SSM	2017	Apr	682	1005	(229, 1720)
SSM	2017	May	1080	1311	(419, 2101)
SSM	2017	Jun	1361	1644	(515, 2752)
SSM	2017	Jul	1100	1329	(418, 2195)
SSM	2018	Apr	949	1157	(351, 1917)
SSM	2018	May	716	871	(286, 1387)
SSM	2018	Jun	1116	1349	(442, 2169)
SSM	2018	Jul	961	1165	(385, 1857)
SSM	2019	Apr	585	723	(182, 1238)
SSM	2019	May	402	564	(125, 1028)
SSM	2019	Jun	467	574	(142, 989)
SSM	2019	Jul	532	649	(178, 1105)
SSM	2020	Apr	798	958	(296, 1708)
SSM	2020	May	599	735	(199, 1234)
SSM	2020	Jun	669	820	(220, 1400)
SSM	2020	Jul	782	951	(267, 1650)
SSM	2021	Apr	752	895	(297, 1528)
SSM	2021	May	913	1108	(328, 1870)

Site	Year	Month	Mean	SD	90% CrI
SSM	2021	Jun	611	744	(217, 1237)
SSM	2021	Jul	419	520	(125, 884)
SSM	2022	Apr	1004	1214	(365, 2039)
SSM	2022	May	816	1000	(293, 1625)
SSM	2022	Jun	1078	1307	(419, 2111)
SSM	2022	Jul	1161	1411	(453, 2310)
