# Supplementary material for: Unraveling abundance from occurrence: Modeling an endangered rodent population with low capture probability
Source: Ecol Appl. 2026 Feb 11;36(1):e70179. doi: 10.1002/eap.70179 (PMC12892172; doi:10.1002/eap.70179)
Supplement: Supplementary file 4 — Appendix S4. [file EAP-36-e70179-s002.pdf]

Unraveling abundance from occurrence: Modeling an endangered rodent population with low capture probability

Ecological Applications

Abby E. Bratt, Cheryl S. Brehme, Robert N. Fisher, Aaron J. Bertoia, Darryl I. MacKenzie

Appendix S4: Additional results, 4-month scale

Table S1: Summary of posterior distributions for capture probability (p) parameters at the long timescale. Given are the posterior mean, standard deviation (SD) and limits of a 90% credible interval (CrI).

Parameter	Mean	SD	90% CrI
μ^p	-2.42	0.19	(-2.73, -2.11)
σ^p	0.26	0.11	(0.08, 0.44)
σ_g^p	0.45	0.17	(0.21, 0.77)
σ_y^p	0.24	0.13	(0.05, 0.47)

Table S2: Summary of posterior distributions for PPM detection probability (ρ) parameters for the long timescale. Given are the posterior mean, standard deviation (SD) and limits of a 90% credible interval (CrI).

Parameter	Mean	SD	90% CrI
μ^ρ	-2.67	0.31	(-3.18, -2.15)
σ^ρ	1.23	0.14	(0.98, 1.45)
β^ρ	0.28	0.42	(-0.40, 0.97)
σ_g^ρ	1.43	0.06	(1.3, 1.5)
σ_y^ρ	0.14	0.11	(0.01, 0.34)

Table S3: Summary of posterior distributions for PPM occupancy probability (ψ) parameters, for the long timescale. Given are the posterior mean, standard deviation (SD) and limits of a 90% credible interval (CrI).

Parameter	Mean	SD	90% CrI
μ^ψ	-1.85	0.38	(-2.46, -1.23)
σ^ψ	1.48	0.02	(1.44, 1.5)
β^ψ	0.59	0.42	(-0.1, 1.29)
σ_g^ψ	1.46	0.04	(1.37, 1.5)
σ_y^ψ	0.80	0.20	(0.51, 1.18)
